# Artificial Intelligence-Based Methodologies for Early Diagnostic Precision and Personalized Therapeutic Strategies in Neuro-Ophthalmic and Neurodegenerative Pathologies

**DOI:** 10.3390/brainsci14121266

**Published:** 2024-12-17

**Authors:** Rahul Kumar, Ethan Waisberg, Joshua Ong, Phani Paladugu, Dylan Amiri, Jeremy Saintyl, Jahnavi Yelamanchi, Robert Nahouraii, Ram Jagadeesan, Alireza Tavakkoli

**Affiliations:** 1 Department of Biochemistry and Molecular Biology, University of Miami Miller School of Medicine, 1600 NW 10th Ave, Miami, FL 33136, USA; rxk641@miami.edu (R.K.); jsaintyl13@gmail.com (J.S.); 2 Department of Clinical Neurosciences, University of Cambridge, Downing Street, Cambridge CB2 3EH, UK; ew690@cam.ac.uk; 3 Department of Ophthalmology and Visual Sciences, University of Michigan Kellogg Eye Center, 1000 Wall St, Ann Arbor, MI 48105, USA; 4 Sidney Kimmel Medical College, Thomas Jefferson University, 1025 Walnut St, Philadelphia, PA 19107, USA; phani.paladugu@students.jefferson.edu; 5 Brigham and Women’s Hospital, Harvard Medical School, 75 Francis St, Boston, MA 02115, USA; 6 Department of Biology, University of Miami, 1301 Memorial Dr, Coral Gables, FL 33146, USA; daa250@miami.edu; 7 Mecklenburg Neurology Group, 3541 Randolph Rd #301, Charlotte, NC 28211, USA; ranahouraii@meckneurology.com; 8 Tandon School of Engineering, New York University, 6 MetroTech Center, Brooklyn, NY 11201, USA; jy4857@nyu.edu; 9 Whiting School of Engineering, Johns Hopkins University, 3400 N Charles St, Baltimore, MD 21218, USA; ramjagad@cisco.com; 10 Human-Machine Perception Laboratory, Department of Computer Science and Engineering, University of Nevada, Reno, 1664 N Virginia St, Reno, NV 89557, USA; tavakkol@unr.edu

**Keywords:** AI-driven ophthalmology, AI-driven therapy, neurodegenerative diseases, machine learning, neural modulation

## Abstract

Advancements in neuroimaging, particularly diffusion magnetic resonance imaging (MRI) techniques and molecular imaging with positron emission tomography (PET), have significantly enhanced the early detection of biomarkers in neurodegenerative and neuro-ophthalmic disorders. These include Alzheimer’s disease, Parkinson’s disease, multiple sclerosis, neuromyelitis optica, and myelin oligodendrocyte glycoprotein antibody disease. This review highlights the transformative role of advanced diffusion MRI techniques—Neurite Orientation Dispersion and Density Imaging and Diffusion Kurtosis Imaging—in identifying subtle microstructural changes in the brain and visual pathways that precede clinical symptoms. When integrated with artificial intelligence (AI) algorithms, these techniques achieve unprecedented diagnostic precision, facilitating early detection of neurodegeneration and inflammation. Additionally, next-generation PET tracers targeting misfolded proteins, such as tau and alpha-synuclein, along with inflammatory markers, enhance the visualization and quantification of pathological processes in vivo. Deep learning models, including convolutional neural networks and multimodal transformers, further improve diagnostic accuracy by integrating multimodal imaging data and predicting disease progression. Despite challenges such as technical variability, data privacy concerns, and regulatory barriers, the potential of AI-enhanced neuroimaging to revolutionize early diagnosis and personalized treatment in neurodegenerative and neuro-ophthalmic disorders is immense. This review underscores the importance of ongoing efforts to validate, standardize, and implement these technologies to maximize their clinical impact.

## 1. Introduction

The early diagnosis and prognosis of neurodegenerative diseases, such as Alzheimer’s disease (AD), Parkinson’s disease (PD), and amyotrophic lateral sclerosis (ALS), as well as neuro-ophthalmic disorders like multiple sclerosis (MS), neuromyelitis optica (NMO), and myelin oligodendrocyte glycoprotein (MOG) antibody disease, represent some of the most pressing challenges in clinical neurology and ophthalmology [1,2,3]. These diseases typically progress insidiously, with subtle signs appearing long before significant clinical manifestations become evident [4,5]. In particular, conditions affecting both the central and peripheral nervous systems, including those impacting the visual pathways and ocular structures, often present with early symptoms such as optic neuritis or visual field defects that are frequently overlooked or misdiagnosed [6,7]. Early detection is critical to slowing or halting disease progression and improving patient outcomes.

Despite significant advancements in diagnostic tools, many of these conditions are diagnosed only after substantial neurological or visual decline has occurred, reducing the effectiveness of treatments [8,9,10]. Thus, identifying reliable biomarkers for early detection remains crucial in enabling timely intervention and improving the prognosis for affected patients [11,12]. Recent advancements in neuroimaging, particularly ultra-high-field magnetic resonance imaging (UHF-MRI) at 7 Tesla (T) and beyond, have significantly enhanced our ability to detect abnormalities with unprecedented precision [13,14]. These cutting-edge imaging systems can identify subtle structural changes, such as hippocampal atrophy in Alzheimer’s disease, nigral degeneration in Parkinson’s disease, or optic nerve atrophy in MS and NMO, long before they become clinically apparent [15,16]. Similarly, advanced diffusion tensor imaging (DTI) allows for the assessment of white matter integrity, revealing early microstructural damage in both brain and optic nerve tissues that previously went undetected [17,18].

These technologies provide critical insights for early diagnosis, but their effectiveness is often limited by the sheer complexity and volume of the data they generate. Artificial intelligence (AI) is revolutionizing the field of neuroimaging by offering sophisticated solutions to this data complexity [19,20,21]. Deep learning algorithms, including convolutional neural networks (CNNs) and transformer-based models, are increasingly capable of performing tasks such as brain and optic nerve segmentation, volume measurement, and pathology identification with exceptional accuracy—often surpassing human capabilities [22,23,24]. AI’s integration with neuroimaging has unlocked new possibilities, particularly through the fusion of multimodal data—combining structural and functional MRI, positron emission tomography (PET), and genetic markers—into unified diagnostic models [25,26]. This approach allows for the early identification of neurodegenerative and neuro-ophthalmic changes in asymptomatic individuals, facilitating earlier interventions and potentially improving patient outcomes [27,28].

Furthermore, the development of next-generation PET tracers targeting amyloid plaques, tau, alpha-synuclein aggregates, and inflammatory markers has provided molecular insights into the pathological processes driving diseases like Alzheimer’s, Parkinson’s, and MS [29,30]. The synergy between advanced neuroimaging techniques and AI analytics enhances diagnostic accuracy and supports the development of personalized treatment strategies tailored to individual patients’ neurobiological profiles [31,32]. While the potential of these technologies is substantial, their clinical implementation is still hindered by challenges such as data heterogeneity, the need for large-scale validation studies, and ethical concerns related to AI-driven systems [33,34,35,36,37,38,39,40]. This review explores the current landscape of advanced neuroimaging and AI technologies, highlighting their potential to transform the early detection, prognosis, and treatment of both neurodegenerative and neuro-ophthalmic disorders, while also addressing the obstacles that must be overcome to integrate these innovations into routine clinical practice.

## 2. Advances in Neuroimaging and AI Integration

I. Diffusion MRI Techniques

Diffusion tensor imaging (DTI) has been widely used for assessing the integrity of white matter [41,42,43,44], providing valuable insights into microstructural changes occurring in neurodegenerative [45,46,47,48,49,50] and associated disorders [51,52]. DTI operates by measuring the anisotropic diffusion of water molecules along axonal fibers, serving as a proxy for the coherence and structural integrity of white matter tracts [53]. However, the limitations of DTI lie in its inability to disentangle overlapping microstructural changes in different tissue compartments, particularly in regions where axonal or cellular complexity is higher, such as in optic nerve and visual pathway structures. This shortfall has driven the evolution of more sophisticated diffusion imaging techniques [54].

Neurite Orientation Dispersion and Density Imaging (NODDI) builds upon DTI by employing a multi-compartment biophysical model to separately quantify neurite density and orientation dispersion [55]. This allows for a more detailed assessment of tissue microarchitecture and has proven especially useful in distinguishing subtle changes within structurally complex regions, such as those impacted by inflammatory and degenerative processes. NODDI enables the isolation of intracellular, extracellular, and isotropic diffusion components, facilitating the identification of early pathological changes. For example, in Parkinson’s disease, NODDI has demonstrated higher sensitivity than DTI in detecting alterations in the substantia nigra, a key motor-related structure, and similar sensitivity may extend to optic radiations and visual cortex regions where early changes can parallel motor-related pathophysiology [56,57,58,59].

Diffusion Kurtosis Imaging (DKI), another advanced diffusion MRI technique, quantifies the non-Gaussian behavior of water diffusion, enabling a more detailed characterization of tissue complexity and heterogeneity [60]. DKI provides metrics such as mean kurtosis (MK), which reflect microstructural complexity and can capture subtle tissue changes not visible through conventional diffusion metrics. In Alzheimer’s and Parkinson’s diseases, DKI has identified early gray matter changes, such as those in the hippocampus or cortical regions, and its sensitivity has similarly been extended to white matter pathways critical for sensory and integrative processing [61,62,63]. The ability of DKI to detect preclinical tissue changes makes it a potential biomarker for monitoring both neural and peripheral axonal integrity.

AI models, particularly machine learning techniques such as random forest classifiers, have been instrumental in leveraging the multidimensional data generated by NODDI and DKI for enhanced diagnostic precision [64]. By integrating neurite density and dispersion metrics with kurtosis-derived features, these algorithms can identify subtle patterns of pathology across brain regions and visual sensory pathways. For instance, studies training models on multimodal diffusion data have achieved up to 94% classification accuracy in distinguishing early-stage Alzheimer’s from healthy controls [65]. Such applications underscore the potential of these imaging modalities when paired with AI to dissect intricate, region-specific changes in diseases with diffuse pathophysiology. The fusion of these advanced techniques highlights their promise in enabling earlier intervention and in offering mechanistic insights into diseases affecting both central and peripheral nervous systems, including the optic nerve and its connections [66].

II. Next-Generation Positron Emission Tomography (PET) Tracers

Positron Emission Tomography (PET) has remained a cornerstone of molecular neuroimaging, offering an unparalleled ability to visualize and quantify the in vivo accumulation of pathological proteins central to neurodegenerative and inflammatory processes [67]. The advent of next-generation PET tracers has markedly enhanced the sensitivity and specificity of PET imaging, particularly in disorders characterized by the aggregation of misfolded proteins such as tau, amyloid-beta, and alpha-synuclein, as well as in conditions marked by neuroinflammatory activity [30,68].

Second-generation tau PET tracers, including 18F-PI-2620 and 18F-MK-6240, have demonstrated superior binding selectivity compared to their predecessors, enabling more accurate quantification of tau pathology [69,70,71]. Tau PET imaging has proven instrumental not only in diagnosing Alzheimer’s disease but also in distinguishing tauopathies from other forms of dementia with sensitivities and specificities exceeding 90% [72,73,74]. The precision of these tracers has implications for detecting subtle tau-related changes, including those within the visual pathways, where tau pathology can manifest in early neurodegenerative or ocular-related conditions. These high-fidelity tracers provide insights into disease progression, particularly when tau pathology begins to affect structures like the visual cortex or optic radiations, enabling earlier intervention [75,76,77].

For synucleinopathies, such as Parkinson’s disease and Lewy body dementia, the development of PET tracers targeting α-synuclein aggregates represents a groundbreaking advancement [78,79,80]. Although still under clinical evaluation, tracers like 11C-MODAG-001 have shown promise in preclinical studies, with the potential to visualize synuclein deposits in regions critical to motor and sensory processing, including structures implicated in visual–motor integration [81,82]. These advancements could pave the way for pre-symptomatic detection of α-synucleinopathies, broadening the applicability of PET imaging to conditions where early sensory or visual deficits emerge [83,84,85].

AI-driven analysis tools, particularly deep learning algorithms, are further enhancing the diagnostic utility of PET imaging by automating and refining image interpretation [86,87,88]. Convolutional neural networks (CNNs), including advanced 3D CNN architectures, have been effectively applied to PET datasets, achieving diagnostic accuracies that rival expert interpretation. For example, AI models analyzing 18F-flortaucipir PET scans for Alzheimer’s detection have reported diagnostic accuracy rates as high as 98.8% [89,90,91,92,93]. These technologies can also facilitate the integration of PET data with structural and diffusion imaging modalities, creating multimodal frameworks capable of capturing early changes in both cortical and subcortical structures, including those relevant to visual processing.

By automating PET scan analysis, AI significantly reduces the reliance on manual interpretation, accelerating clinical workflows and enabling more widespread adoption of PET imaging [28,94,95]. This is particularly relevant for conditions where dynamic or multimodal imaging is required to monitor disease progression or response to targeted therapies. The integration of PET imaging with advanced AI systems holds tremendous potential for unraveling complex pathologies that affect interconnected brain and sensory systems, enabling personalized diagnostic and therapeutic approaches.

III. Multi-Modal Integration

One of the most transformative advancements in the application of AI to neuroimaging is its ability to integrate data from multiple imaging modalities—such as structural MRI, functional MRI (fMRI), PET, and genetic data—into unified diagnostic frameworks [96,97]. This multimodal approach provides a comprehensive understanding of diseases that affect interconnected systems, such as neurodegenerative and neuro-ophthalmic disorders, by capturing structural, functional, and molecular insights that single-modality imaging often misses [19,98]. For example, in diseases like MS, NMO, and MOG antibody disease, where the visual system is frequently involved, such integration enables the detection of both central and peripheral pathology. Transformer-based AI models, such as the Vision Transformer (ViT), have demonstrated remarkable utility in processing multimodal neuroimaging data [99,100]. While inspired by the transformer architecture originally developed for natural language processing, ViTs were specifically designed for image-based tasks and have been successfully adapted for 3D medical imaging. This adaptation enables ViTs to capture long-range dependencies within neural and visual structures [101]. For multimodal integration, the use of standardized brain map structures, such as MNI or Talairach templates, is essential and commonly employed in neuroimaging studies to align and normalize data from various modalities. This ensures consistency in analyzing regions like the optic radiations, lateral geniculate nucleus (LGN), and visual cortex while mitigating variability due to differences in patient positioning or scanningprotocols. This also reduces variability due to differences in patient positioning and scanning protocols, allowing AI models to more accurately detect subtle alterations associated with early inflammatory or degenerative changes. For example, a study using 3D ViT models to classify Alzheimer’s disease from healthy controls achieved an accuracy of 96.7%, outperforming traditional CNN-based models [102]. In neuro-ophthalmic applications, ViT models can analyze the optic radiations, lateral geniculate nucleus (LGN), and visual cortex, detecting subtle alterations associated with early inflammatory or degenerative changes.

Multimodal Transformer networks, such as the Multimodal Transformer for Alzheimer’s Disease (MT-AD), similarly exemplify the power of integrating diverse datasets. The MT-AD framework fuses data from structural MRI, fMRI, PET imaging, and genetic profiles, achieving a remarkable area under the receiver operating characteristic curve (AUC) of 0.98 in early Alzheimer’s detection [25,94]. In neuro-ophthalmic disorders, this integration could detect disruptions in the visual pathway, including optic nerve damage (as seen in MS, NMO, and MOG antibody disease) or cortical involvement in the visual cortex, which may precede overt clinical symptoms. By capturing cross-modal patterns of structural degeneration and metabolic changes, AI systems can identify early biomarkers in both the brain and the visual system [90,92,93].

Graph Neural Networks (GNNs) further enhance multimodal analysis by modeling connectivity patterns across brain networks [103,104]. The Brain Connectivity Graph Neural Network (BC-GNN) integrates data from MRI, PET, and diffusion tensor imaging (DTI) to map functional and structural networks with exceptional precision [105]. For example, BC-GNN has been shown to distinguish early-stage Parkinson’s disease from healthy controls with an accuracy of 92% [106,107]. When applied to neuro-ophthalmic diseases, GNNs could reveal disruptions in connectivity between the visual cortex, LGN, and other sensory processing regions. This approach enables the identification of subtle connectivity changes that traditional imaging methods might miss, particularly in diseases where the visual system serves as a critical early indicator of broader neurological dysfunction.

The integration of multimodal imaging, driven by AI, holds significant potential for transforming the diagnosis and management of neuro-ophthalmic disorders (Table 1). To ensure consistency across modalities, spatial normalization techniques and uniform brain map structures, such as MNI or Talairach templates, are crucial. For example, in MS, the combination of structural MRI to assess optic nerve integrity, fMRI to evaluate visual cortex activity, and PET to detect inflammatory markers along the visual pathway benefits from the alignment of all modalities to a common brain template. This unified framework enhances the reliability of the integrated data, providing a clearer understanding of disease progression.

IV. Translational Impact

The clinical translation of these advanced neuroimaging and AI technologies is rapidly advancing, with significant implications for the diagnosis, prognosis, and treatment of neurodegenerative diseases [31,32]. The integration of AI-driven systems with neuroimaging has the potential to revolutionize not only early detection but also personalized medicine in neurology (Table 2), offering a more tailored and effective approach to patient care [33,34]. One notable example of translational research is the development of adaptive deep brain stimulation (DBS) systems for Parkinson’s disease [108,109]. Traditional DBS therapy uses a fixed stimulation pattern, which can provide symptomatic relief but does not account for the dynamic nature of the disease [110]. Adaptive DBS systems leverage real-time feedback from neuroimaging and AI algorithms to adjust stimulation parameters based on the patient’s current brain state [111,112]. By continuously monitoring brain activity and adjusting the stimulation in real time, these systems can optimize therapeutic effects, improving motor function while reducing side effects such as dyskinesia [113,114]. Some studies have reported up to a 30% improvement in motor symptoms when using adaptive DBS systems compared to conventional methods [115]. AI-enhanced retinal imaging is another area where significant advancements are being made in the non-invasive detection of neurodegenerative diseases [45,48]. Optical coherence tomography (OCT) imaging of the retina has gained traction as a low-cost, easily accessible method for identifying early neurodegenerative changes [47,116]. Machine learning algorithms trained on OCT images can detect subtle changes in retinal morphology that correlate with brain pathology, offering a potential diagnostic tool for early-stage disease detection [117,118]. Studies have reported sensitivities and specificities exceeding 90% for detecting early-stage Alzheimer’s disease (AD) and Parkinson’s disease (PD) using AI models applied to OCT data [119,120].

V. Privacy and Data Sharing

Further advancements in federated learning have the potential to address the privacy and data-sharing challenges inherent in medical research, particularly in neuroimaging studies [121,122]. Federated learning enables AI models to be trained on decentralized datasets across multiple institutions without the need to centralize sensitive patient data [123]. This approach could accelerate the development of AI-driven diagnostic tools by providing access to larger, more diverse datasets while maintaining patient privacy [124,125]. Federated learning has already shown promise in enabling collaborative research across medical institutions and could be particularly valuable for creating AI models capable of generalizing across diverse populations and clinical settings [126,127]. Another emerging frontier is the integration of neuroimaging biomarkers with liquid biopsy techniques, such as blood-based assays for amyloid-β, tau, and other neurodegenerative proteins [128,129]. Combining neuroimaging data with molecular markers from blood samples could provide a more comprehensive, non-invasive method for diagnosing and tracking disease progression [130,131]. AI models could be used to integrate these data streams, enhancing their predictive power and enabling a more personalized approach to monitoring disease progression [132,133]. Early detection through blood biomarkers, combined with neuroimaging, holds great potential for detecting diseases like Alzheimer’s and Parkinson’s in their pre-symptomatic stages, allowing for earlier interventions that may slow disease progression [134,135].

The integration of AI-driven multimodal imaging systems with advanced neurophysiological monitoring also opens up new possibilities for the real-time assessment of brain function in clinical settings [136,137]. For example, coupling structural and functional MRI with electrophysiological data from techniques such as electroencephalography (EEG) or magnetoencephalography (MEG) could provide real-time insights into brain activity and connectivity [138,139]. AI models trained on these multimodal data could detect changes in brain networks that precede clinical symptoms, allowing for early intervention and potentially slowing the onset of neurodegenerative diseases [140,141].

VI. Impact on Neurorehabilitation

Beyond diagnosis and monitoring, these technologies also have great potential to transform neurorehabilitation. For example, functional MRI (fMRI) can be used to track changes in brain activity as patients engage in rehabilitative tasks [142,143,144]. When combined with real-time AI analysis, fMRI data could guide adaptive neurorehabilitation strategies, providing personalized feedback to patients and clinicians to enhance recovery [145,146]. Similarly, AI-driven analysis of diffusion MRI could identify regions of the brain that are most vulnerable to neurodegeneration, allowing for targeted rehabilitative interventions aimed at preserving brain function [147,148]. Further, while transcranial stimulation, including techniques like transcranial magnetic stimulation (TMS) and transcranial direct current stimulation (tDCS), has shown promise in modulating brain activity, its application in AI-directed neurostimulation remains an emerging field [149,150]. Current studies focus on using AI to optimize stimulation parameters, but direct evidence supporting its clinical efficacy in neurodegenerative diseases is limited. Further research is needed to validate these methods in controlled settings before widespread adoption [151,152,153].

## 3. Challenges in Integrating AI into Clinical Practice for Diagnosis and Treatment

The application of AI faces significant technical challenges, primarily due to the inherent variability of neuroimaging data [21,35,154,155,156,157]. This variability stems from multiple sources, such as differences in MRI scanners, imaging protocols, and patient positioning, all of which introduce inconsistencies that can impact AI model accuracy and generalizability [158]. To mitigate position-related challenges, the use of uniform brain map structures, such as MNI or Talairach templates, is critical and widely adopted in neuroimaging studies. These standardized coordinate systems ensure consistent alignment of brain regions across scans and subjects, even when using the same scanner. By applying spatial normalization techniques based on these templates, positional variability is significantly reduced, enhancing the reliability of AI models and supporting cross-study comparisons in diverse clinical settings.

## 4. Technical Challenges: Variability in Neuroimaging Data and Its Impact on AI Models

Scanner variability is a key challenge in neuroimaging, particularly when comparing MRI systems with different field strengths (e.g., 1.5 T vs. 3 T) [159,160,161,162,163,164]. Even within the same manufacturer, MRI scanners can exhibit variations in image characteristics due to differences in gradient performance [121,122,154,165,166], radiofrequency coil designs [155,163,164,165,167,168,169], and reconstruction algorithms [35,164,165,170,171,172,173]. These physical differences in field strength and hardware configurations can result in inconsistencies in image contrast, resolution, and signal-to-noise ratio, complicating the application of AI algorithms trained on data from specific scanner types [174]. While advancements in scanner technology can reduce certain artifacts, the fundamental physical changes associated with varying field strengths cannot be completely eliminated through manufacturing strategies alone. These inherent differences must be accounted for during data analysis to maintain accurate and consistent AI model performance.

Protocol variability presents a significant challenge in neuroimaging. Imaging protocols can vary significantly between institutions and even within the same institution over time. Differences in pulse sequences, acquisition parameters (such as repetition time [TR], echo time [TE], and flip angle), contrast agent usage, and image plane orientation contribute to disparities in tissue contrasts and artifact patterns [175]. These variations can negatively impact the generalizability of AI models, especially when trained on data from specific protocols that may not perform well across a range of imaging settings. However, the use of mapping data, such as standardized brain templates or spatial normalization techniques, can significantly minimize these protocol-related issues. By aligning data from different scanners and protocols to a common coordinate system, mapping data ensures consistency across diverse imaging protocols, improving the reliability and generalizability of AI models in clinical applications [176].

Patient positioning and motion during scans introduce significant challenges in neuroimaging, as variations in head orientation, motion artifacts, and physiological noise (e.g., respiratory and cardiac cycles) can cause distortions and misalignments in images, potentially compromising AI model performance [177,178]. However, the use of a uniform brain map structure—such as standardized brain atlases (e.g., MNI or Talairach)—can help mitigate these challenges. By applying spatial normalization techniques, individual brain scans can be aligned to a common template, reducing the variability caused by differences in patient positioning. Advanced registration algorithms further correct for head position differences, ensuring consistent alignment across subjects and scans. Additionally, probabilistic atlases and statistical parametric mapping (SPM) can account for inter-subject variability, providing a more robust framework for analyzing neuroimaging data. These approaches, when integrated with AI models, enhance the accuracy and reliability of neuroimaging analysis in clinical settings, helping to overcome the inevitable variability inherent in patient positioning and motion during scans [178].

Another source of variability arises in image preprocessing. Different preprocessing pipelines used to prepare neuroimaging data for analysis can introduce inconsistencies in the final images. Techniques such as bias field correction, skull stripping, registration, normalization, and intensity standardization can all vary, potentially affecting AI model performance if not consistently applied [179,180].

Longitudinal changes present additional challenges in studies tracking disease progression over time. Factors such as scanner upgrades, protocol updates, or patient-specific changes unrelated to the disease being studied can introduce confounding variables that AI models must account for in order to accurately track disease progression [181].

Finally, multi-site data integration introduces complexities that AI models must overcome to ensure their robustness. Data from different sites can differ in terms of patient demographics, clinical assessment protocols, and data quality control measures. Such site-specific biases can hinder the generalizability of AI models, necessitating careful strategies to ensure that models are effective across diverse clinical environments [182,183].

- Regulatory and Legal Barriers: Ensuring Compliance and Addressing Liability

These technical challenges directly tie into regulatory concerns. The U.S. Food and Drug Administration (FDA) requires substantial evidence that AI-based diagnostic devices maintain performance across diverse conditions, including variations in scanner types, protocols, and patient populations [184,185]. This requirement poses significant hurdles for AI developers, as they must demonstrate the ability of their models to remain reliable and accurate across different clinical settings. The FDA requires AI models to be validated under regulatory pathways such as the 510(k) pathway or Premarket Approval (PMA) processes [171,186]. For example, AI models must be robust to scanner variability, requiring validation studies using data from multiple scanner models and manufacturers to ensure consistent performance [49]. Similarly, AI models must be able to handle protocol variability, potentially requiring the development of protocol-agnostic architectures or extensive fine-tuning capabilities to maintain performance across different imaging protocols [187]. AI systems may also need to demonstrate resilience to motion artifacts, possibly requiring the integration of motion correction algorithms or the ability to flag highly motion-corrupted images for manual review [188]. Furthermore, the FDA may mandate preprocessing standardization or require models to be robust to variations in preprocessing methods, which could drive the development of industry-wide standards for neuroimaging AI applications [189]. For AI systems designed to track disease progression, the FDA may require evidence of longitudinal consistency, ensuring that the model performs effectively despite scanner upgrades, protocol changes, or other temporal variations [190]. Lastly, the FDA is likely to require validation of AI model performance across multiple sites and diverse patient populations to ensure multi-site generalizability, which may involve extensive multi-center validation studies [158].

Liability for misdiagnosis due to image variability is also a primary concern. If an AI system misdiagnoses a patient because of such variability, questions about responsibility arise. Determining liability—whether it lies with the AI developer, healthcare provider, or imaging facility—may require new legal frameworks to address AI-assisted diagnoses [169]. Additionally, data sharing and privacy are critical concerns. Sharing medical imaging data across institutions is necessary to develop robust AI models, but it raises privacy issues and must comply with regulations like HIPAA in the United States and GDPR in the European Union. This creates a tension between the need for diverse training datasets and the protection of patient privacy rights [122]. Informed consent also becomes a crucial issue. Patients must be informed that AI systems are being used in their diagnosis, including the potential for errors due to variability in the data. Communicating the risks and benefits of AI-assisted diagnosis to patients is a legal challenge in itself [35]. Furthermore, existing FDA regulatory approval pathways, such as the 510(k) and PMA processes, may not be well-suited to the unique characteristics of AI systems that continuously learn and adapt. New regulatory frameworks may be necessary to address these concerns [186]. Post-market surveillance is another area where traditional methods may be inadequate. AI systems that evolve over time must be monitored to ensure they do not introduce new risks or errors, which may require new approaches to post-market monitoring and regulation [171]. Finally, international harmonization of regulations becomes increasingly important as AI systems are deployed globally. Different regulations across countries regarding medical AI and data privacy could complicate the widespread adoption of AI tools, necessitating efforts toward international regulatory alignment [164].

II. Future Directions: Addressing Variability Through AI Innovations

Several potential solutions and future directions are being explored to address the challenges currently faced in the application of artificial intelligence (AI) in healthcare and neuroimaging (Table 3). One promising approach is adaptive neuroimaging harmonization (ANH), which utilizes transfer learning and domain adaptation techniques to mitigate issues related to scanner and protocol variability. While ANH holds great promise, regulatory concerns remain regarding how these adaptive techniques fit into current clinical frameworks [191,192,193]. Another important direction is federated learning, which enables AI models to be trained on distributed datasets without centralizing sensitive patient data. This approach addresses privacy concerns while maintaining access to diverse training data, ensuring that models are robust and applicable across varied patient populations [121,168,194,195].

As AI models become increasingly integrated into healthcare settings, Explainable AI (XAI) has emerged as a critical component for the adoption and acceptance of AI technologies. The “black box” nature of many AI models, particularly deep learning systems, has raised concerns over transparency and accountability in clinical practice. XAI addresses this challenge by providing mechanisms to interpret and understand AI model predictions, ensuring that clinicians can trust AI-generated decisions and integrate them confidently into patient care. A key challenge in XAI development is to provide clinically relevant, interpretable models that allow healthcare professionals to understand how AI systems arrive at their conclusions. Local interpretability methods, such as LIME (Local Interpretable Model-agnostic Explanations) and SHAP (SHapley Additive exPlanations), enable the generation of explanations that highlight the key features influencing AI predictions. These techniques have demonstrated success in enhancing the transparency of AI models applied to complex neuroimaging tasks, such as brain tumor detection and early diagnosis of neurodegenerative diseases.

Moreover, XAI is increasingly being integrated with advanced models that combine rule-based systems, gradient-based techniques, and attention mechanisms. These hybrid frameworks offer a more nuanced approach to explaining AI decisions, enabling clinicians to gain deeper insights into the reasoning behind each prediction. Such developments are particularly valuable in medical imaging, where accurate and interpretable results are critical for clinical decision-making. The application of XAI is especially important in neuroimaging, where AI systems must demonstrate transparency to gain regulatory approval and clinician acceptance. The integration of domain knowledge and expert input into XAI frameworks will help ensure that AI tools remain aligned with clinical workflows and are trusted by medical professionals. Furthermore, as AI becomes more embedded in healthcare applications like AI of Medical Things (AIoMT), XAI will play a key role in ensuring that these systems are both reliable and understandable.

Looking ahead, several challenges remain for XAI in healthcare. One significant hurdle is balancing the complexity of AI models with their interpretability, ensuring that explanations do not oversimplify but still remain clinically relevant. Establishing standardized metrics for evaluating the quality and effectiveness of XAI explanations is another crucial task, as it will make it easier to compare different approaches and select the most suitable ones for specific clinical applications. Additionally, ensuring that XAI methods can be effectively integrated into clinical workflows is essential, providing explanations that are actionable and enhance decision-making processes. Mitigating bias in AI models through explainability is also a critical concern, as it helps ensure fair and equitable outcomes across diverse patient populations.

In conclusion, the development of explainable AI is essential for the continued adoption of AI in healthcare. By providing transparency, improving model trustworthiness, and ensuring that AI tools align with clinical practices, XAI will be crucial in overcoming the regulatory, ethical, and practical challenges of implementing AI technologies in neuroimaging and healthcare. As XAI continues to evolve, it will enable AI to make a significant impact in precision medicine, ensuring that these technologies are both effective and ethical in their application. The ongoing research and development in XAI will not only enhance the interpretability of AI models but also foster greater trust and collaboration between AI systems and healthcare professionals, ultimately leading to improved patient outcomes and more efficient healthcare delivery.

## 5. Discussion

The convergence of advanced neuroimaging and AI in the diagnosis and treatment of neurodegenerative diseases has the potential to significantly alter the clinical landscape. However, while the promise of these technologies is vast, their integration into routine clinical practice presents both opportunities and challenges that require careful consideration.

At the heart of this transformation is the ability to detect subtle, early changes in the brain that were previously undetectable. Advanced imaging techniques like diffusion MRI (e.g., NODDI and DKI) offer clinicians a powerful tool to observe microstructural alterations before cognitive or motor symptoms appear. Early detection could mean that interventions are started at a point where they are more effective, potentially delaying disease progression or improving quality of life. This shift from reactive to proactive healthcare is a paradigm shift that clinicians can take advantage of, allowing for more targeted, timely interventions. The real challenge for clinicians, however, is in translating these early imaging findings into actionable clinical decisions.

The real-world application of these technologies goes beyond simply diagnosing disease earlier; it is about enhancing the precision and personalization of treatment. Imagine a scenario where a clinician is able to combine advanced neuroimaging findings with AI-driven insights to not only detect the disease but also predict its trajectory based on individual brain structure and function. AI tools can help clinicians identify the subtle distinctions in disease progression that might otherwise go unnoticed, allowing for more personalized therapeutic approaches. For example, AI-enhanced models could suggest whether a patient with early Alzheimer’s is likely to experience rapid cognitive decline or whether their disease might remain relatively stable for an extended period. This level of precision allows for more informed treatment plans and the ability to tailor interventions to each patient’s unique presentation, ensuring that patients receive the most appropriate care based on their specific needs.

While the potential for personalized care is tremendous, the integration of these advanced technologies into clinical practice is not without its challenges. One of the most significant hurdles is ensuring that clinicians can trust and understand the outputs of AI models. AI systems excel at processing complex data and detecting patterns, but the clinical decision-making process requires context—an understanding of the patient’s full medical history, symptoms, and personal circumstances. AI can provide invaluable insights, but clinicians must remain at the forefront of the decision-making process, using AI as a tool to augment their expertise, not replace it. The integration of AI into clinical practice should not lead to a dehumanization of care, where patients become data points in a system, but rather should enhance the clinician’s ability to make well-informed, empathetic decisions.

Moreover, the successful use of AI and neuroimaging in clinical practice requires clinicians to have the appropriate training and support. AI systems are powerful, but they are only as effective as the people using them. The current medical education system, which primarily trains clinicians in traditional methods of diagnosis and treatment, must evolve to ensure that healthcare providers are equipped with the skills to work alongside advanced AI technologies. This could mean more emphasis on data literacy, AI ethics, and the interpretation of complex neuroimaging data as part of medical training. Additionally, clinicians must have the infrastructure and tools necessary to integrate these technologies into their daily workflows. This includes having access to AI-driven diagnostic tools, real-time neuroimaging feedback, and seamless systems for managing and interpreting multimodal data.

Another key consideration is the ethical and regulatory landscape. The widespread use of AI in healthcare raises questions about data privacy, algorithmic bias, and accountability. Who is responsible if an AI system makes an error in diagnosis or treatment? What safeguards are in place to protect patient data when it is processed by machine learning models? These are critical questions that clinicians must be prepared to answer as they begin to incorporate AI into their practice. Furthermore, as AI models are continuously trained on new data, they may evolve in ways that are not immediately predictable. This raises concerns about the long-term reliability and consistency of AI systems. Clinicians need to feel confident that the systems they rely on are safe, transparent, and governed by ethical principles that prioritize patient welfare.

## 6. Conclusions

Looking to the future, the combination of AI and advanced neuroimaging offers a vision of personalized medicine that was once unimaginable. It holds the potential to revolutionize not only how we diagnose and treat neurodegenerative diseases, but also how we think about patient care as a whole. The ability to tailor treatments to individual patients, predict disease progression, and intervene at earlier stages could significantly alter the course of neurodegenerative diseases. However, for this vision to be realized, several challenges must be overcome.

While AI’s potential in healthcare is vast, its integration into clinical practice requires careful attention to several critical issues. Data limitations, including the need for large, high-quality, and diverse datasets, remain a significant challenge. AI models rely on comprehensive datasets to provide accurate predictions, but these datasets are not always available, particularly in underserved or underrepresented patient populations. Additionally, bias in AI models, arising from skewed or unrepresentative data, can exacerbate existing healthcare disparities, making it essential to address fairness in data collection and model training.

Another key challenge is the interpretability of AI models. Many AI systems, especially those based on deep learning, function as “black boxes”, where even experts cannot easily understand how a decision was made. This lack of transparency raises concerns about the trustworthiness of AI-generated diagnoses. For AI to gain widespread adoption in healthcare, it is critical to develop explainable AI (XAI) frameworks that allow clinicians to interpret AI predictions and make informed decisions based on these insights. Explainability is not only necessary for clinical confidence but also for ensuring regulatory compliance and ethical accountability in AI-assisted decision-making.

Moreover, regulatory hurdles remain a significant barrier to the widespread deployment of AI in clinical settings. AI systems must meet rigorous standards before they can be integrated into healthcare workflows, and these standards must ensure that AI tools are both effective and safe for patient care. Addressing these regulatory challenges will require ongoing collaboration between AI developers, healthcare providers, and regulatory bodies.

Ethical concerns regarding data privacy and patient consent also need to be resolved. As AI systems rely on large amounts of patient data, ensuring that data is handled securely and ethically is crucial. This involves maintaining compliance with privacy regulations (such as GDPR and HIPAA) while balancing the need for diverse data to train robust AI models.

In light of these challenges, the future success of AI in healthcare depends on collaboration between clinicians, researchers, and AI developers. Only by working together can we ensure that AI technologies are implemented in ways that enhance, rather than disrupt, patient care. This collaborative effort will not only address the technical and ethical challenges AI presents but also promote a patient-centered approach, ensuring that AI tools support clinicians in their efforts to provide the best possible care.

Ultimately, the integration of AI and advanced neuroimaging into clinical practice requires a balanced approach—one that combines technological innovation, clinical expertise, ethical oversight, and patient-centered care. By addressing the challenges of data quality, interpretability, bias, and regulatory compliance, we can ensure that AI not only improves diagnostic accuracy and treatment outcomes but also fosters greater trust and transparency. Embracing these tools thoughtfully and carefully will pave the way for a new era of personalized, data-driven healthcare, ensuring that AI is used responsibly to improve patient outcomes and transform the healthcare landscape.

## Figures and Tables

**Table 1:** Summary of AI techniques, disorders investigated, methods proposed, and results obtained.

AI Technique	Disorders Investigated	Methods Proposed	Results Obtained
Neurite Orientation Dispersion and Density Imaging (NODDI)	Alzheimer’s Disease, Parkinson’s Disease, Multiple Sclerosis, Myelin Oligodendrocyte Glycoprotein Antibody Disease	Diffusion MRI to assess microstructural changes in brain and visual pathways.	Enhanced sensitivity in detecting early-stage neurodegeneration and microstructural changes in complex regions like substantia nigra and optic radiations.
Diffusion Kurtosis Imaging (DKI)	Alzheimer’s Disease, Parkinson’s Disease, Multiple Sclerosis, Neuromyelitis Optica	Advanced diffusion MRI to quantify non-Gaussian water diffusion, assessing tissue heterogeneity.	Early detection of subtle gray and white matter changes in cortical and hippocampal regions; potential biomarker for neurodegenerative diseases.
Convolutional Neural Networks (CNNs)	Alzheimer’s Disease, Parkinson’s Disease, Multiple Sclerosis, Neuromyelitis Optica	Deep learning model applied to multimodal MRI and PET imaging data.	Achieved diagnostic accuracy of up to 98.8% in distinguishing Alzheimer’s from healthy controls using 18F-flortaucipir PET scans.
Multimodal Transformers	Alzheimer’s Disease, Parkinson’s Disease, Myelin Oligodendrocyte Glycoprotein Antibody Disease, Multiple Sclerosis	Integration of multimodal imaging data (MRI, PET) and genetic data for disease prediction.	Excellent AUC (0.98) in early Alzheimer’s detection, revealing potential to identify disruptions in the visual pathway and cortical involvement.
Graph Neural Networks (GNNs)	Parkinson’s Disease, Multiple Sclerosis, Myelin Oligodendrocyte Glycoprotein Antibody Disease	Network modeling of brain regions using multimodal data to understand connectivity and disease progression.	Distinguished early-stage Parkinson’s disease from healthy controls with 92% accuracy, identifying disruptions in connectivity across sensory processing regions.

**Table 2:** Summary of key neuroimaging techniques and AI integration in clinical practice for disease diagnosis and treatment.

Technology	Details
Diffusion Tensor Imaging (DTI)	Provides insights into white matter integrity, useful for detecting disease progression in neurodegenerative diseases. However, limited in differentiating microstructural changes in tissue.
Neurite Orientation Dispersion and Density Imaging (NODDI)	Enhances the detection of early microstructural changes in diseases like Parkinson’s. Offers more nuanced views of white matter integrity. Requires specialized equipment and computational power.
Diffusion Kurtosis Imaging (DKI)	Detects non-Gaussian diffusion behavior and identifies early gray matter changes in diseases such as Alzheimer’s. Clinically relevant but requires sophisticated analysis tools.
AI Integration with NODDI and DKI	AI improves diagnostic accuracy by analyzing complex data, enabling early detection with higher precision (e.g., 94% accuracy for Alzheimer’s detection). Models require continuous validation.
Tau PET Tracers (e.g., 18F-PI-2620)	Enhances specificity and sensitivity for tau imaging, crucial for early Alzheimer’s diagnosis. Challenges include availability and cost.
Alpha-Synuclein PET Tracers (e.g., 11C-MODAG-001)	Key for diagnosing synucleinopathies like Parkinson’s at earlier stages. Still in clinical trials, but offers the potential to detect disease before symptoms appear.
AI-driven PET Imaging (CNNs, 3D CNN Architectures)	AI systems automate PET scan analysis, reducing manual interpretation time and improving diagnostic consistency. Requires transparency for trust and clinical integration.
Transformer-based AI Models (e.g., Vision Transformer)	AI models like ViT capture long-range dependencies in 3D data, improving diagnostic accuracy for conditions like Alzheimer’s. Need for large, high-quality datasets and computational resources.
Graph Neural Networks (GNNs) for Brain Connectivity	GNNs model brain connectivity disruptions, critical for early-stage Parkinson’s diagnosis. Requires high data consistency and overcoming complex integration barriers.
Adaptive Deep Brain Stimulation (DBS)	AI enhances DBS by adjusting stimulation in real time, improving therapeutic outcomes for Parkinson’s patients. Challenges include regulatory approval and monitoring for continuous adaptation.
AI-enhanced Retinal Imaging (Optical Coherence Tomography—OCT)	Provides a non-invasive, cost-effective method for detecting early-stage Alzheimer’s and Parkinson’s by identifying retinal changes linked to brain pathology. Still not universally adopted.
Neuroimaging Biomarkers + Liquid Biopsy	Combining neuroimaging with blood biomarkers offers a comprehensive, non-invasive approach for monitoring disease progression and enabling early interventions. Requires data integration strategies.

**Table 3:** Main challenges clinicians face when integrating AI technologies into diagnosis and treatment.

Challenge	Details
Variability in Imaging Data	Variations in MRI scanners, protocols, patient positioning, and preprocessing pipelines create inconsistencies that impact the accuracy and generalizability of AI models. This affects their performance across diverse clinical settings.
Scanner and Protocol Differences	Even within the same manufacturer, MRI scanners may produce different image characteristics (e.g., field strength, coil design), leading to variability in diagnostic accuracy. Variability in imaging protocols across institutions further complicates AI’s ability to generalize and maintain consistent performance.
Patient Positioning and Motion	Variations in patient positioning and involuntary motion (e.g., respiratory, cardiac cycles) introduce distortions that can negatively affect AI-driven diagnoses. AI models must account for such variability in real-time to maintain diagnostic accuracy.
Image Preprocessing Variability	Differences in preprocessing steps (e.g., skull stripping, registration, normalization) can lead to inconsistencies in the final image data, potentially hindering AI performance if not standardized across clinical settings.
Longitudinal Variability	Changes in imaging technology, protocols, and patient-specific factors over time complicate longitudinal tracking of disease progression. AI models must adapt to these changes to remain effective in monitoring disease development.
Multi-Site Data Integration	Data from different clinical sites can vary due to differences in patient demographics, protocols, and quality control measures. AI models must be robust enough to handle these variations to ensure they work across a wide range of clinical environments.
Regulatory Compliance	The FDA requires rigorous validation of AI models to ensure they perform reliably across diverse clinical conditions. Models must be validated under appropriate regulatory pathways, such as 510(k) or PMA, while also adapting to evolving scanner technology and clinical protocols.
Legal Liability	AI-driven misdiagnosis due to image variability raises liability questions about who is responsible (AI developer, healthcare provider, or imaging facility). New legal frameworks may be needed to clarify liability for AI-assisted diagnoses.
Data Privacy and Security	Sharing medical imaging data across institutions is essential for developing robust AI models, but it raises concerns over patient privacy. Adhering to HIPAA, GDPR, and other data protection regulations creates tension between the need for diverse training data and safeguarding privacy.
Informed Consent	Patients must be informed about the role of AI in their diagnosis and the potential risks of errors due to data variability. Clearly communicating the benefits and limitations of AI-assisted diagnoses is a critical ethical and legal issue.
Regulatory Gaps for AI Adaptation	Current FDA regulatory pathways may not fully accommodate the continuous learning nature of AI systems, raising the need for new regulatory frameworks to address the evolving nature of AI models.
Post-Market Surveillance	AI systems that adapt over time require continuous monitoring to ensure they do not introduce new risks or errors. Traditional post-market surveillance methods may not be suitable for AI’s dynamic nature, necessitating the development of new approaches.
International Regulatory Alignment	Different regulations regarding medical AI and data privacy across countries complicate global adoption. Efforts toward international regulatory harmonization are necessary to facilitate widespread deployment and ensure patient safety.
